# Supplementary material for: Defecting or Not Defecting: How to “Read” Human Behavior during Cooperative Games by EEG Measurements
Source: PLoS One. 2010 Dec 1;5(12):e14187. doi: 10.1371/journal.pone.0014187 (PMC2995728; doi:10.1371/journal.pone.0014187)
Supplement: Methods S1 — (0.02 MB DOC) [file pone.0014187.s001.doc]

# Methods S1

### ANOVA (Analysis Of Variance)

We adopted a repeated-measures ANOVA statistical design for each separate frequency band since the oscillating properties of the brain generally show independent results and can be considered as separate frequency channels. The dependent variables were the 52 total strength values (*s_in_ + s_out_*) obtained from each couple of subjects (26 couples) by considering the contribution of the sub-networks of the two brains composing the hyper–brain. Two independent factors were taken into account in the ANOVA: the TASK factor, with three levels (i.e. the experimental situations CC, DD and TT) and the ROI factor with six levels (i.e. the considered cortical areas). Subsequently, Duncan’s post-hoc tests were used to reveal which cortical region presented the highest significant variations within the experimental conditions. The significance level was set at p=0.001 for both the ANOVA and post-hoc analyses. Greenhouse & Geisser correction was used for protection against the violation of the sphericity assumption in the repeated measure ANOVA. All of the statistical analysis was performed with the software Statistica©, StatSoft, Inc.
