# Supplementary material for: Defecting or Not Defecting: How to “Read” Human Behavior during Cooperative Games by EEG Measurements
Source: PLoS One. 2010 Dec 1;5(12):e14187. doi: 10.1371/journal.pone.0014187 (PMC2995728; doi:10.1371/journal.pone.0014187)
Supplement: Methods S2 — (0.03 MB DOC) [file pone.0014187.s002.doc]

# Methods S2

### Multi–layer perceptron

The MultiLayer Perceptron (MLP) [S11; S12] is an artificial non–linear neural network, which maps a subset of an *N*–dimensional input space onto a subset of an *M*–dimensional output space. Each neuron in a MLP receives a certain number of inputs and produces an output using a non–linear activation function, usually a sigmoidal function such as the logistic function or the hyperbolic tangent function. The output of a neuron is obtained by computing the value of the activation function corresponding to the sum of all its inputs, which is roughly the same mechanism exploited by human neurons to produce electrical signals and to send them to neighbors.

Neurons of a MLP are organized in layers, so that all neurons of a layer receive inputs from neurons in the previous layer and provide inputs to the neurons of the next layer. The connections among neurons are represented by weighted “synapses”: each synapse connects the output of a neuron in a layer to an input of another neuron in the next layer. A schematic representation of a MLP is reported in **Figure S5**.

The leftmost layer is called *Input Layer* while the rightmost one (i.e. the last layer) is called *Output Layer*. All layers between the input layer and the output layer are called *Hidden Layers*. Note that the value of the output of a neuron in layer *k* depends, through the non–linear activation function, only on the sum of inputs received from the neurons in layer *k*−1, which are, in turn, computed using inputs from layer *k*−2 and so on, up to the input layer.

The feature that makes MLPs interesting for practical use is that they are able to “learn” a certain mapping of inputs into outputs. It means that there is a supervised learning algorithm, called “Back–propagation”, that iteratively changes the weights of synapses, which connect neurons in order to minimize the error between the actual output of the network and the one provided as a sample.

In order to “teach” an MLP the mapping between a certain input space and an output space, it is necessary to have a set of “patterns”, i.e. couples , which represent an *N*–dimensional input *x* and the corresponding expected *M*–dimensional output *y*. Then, the input vector of each pattern is fed to the network and the obtained output is compared to the desired one. Given an output error, the back–propagation algorithm iteratively adjusts the weights of the synapses in order to minimize the error, and then a new pattern can be submitted to the MLP. We say that the MLP has “learned” a mapping if the mean square error over all the set of patterns is below a preset threshold, which depends on the application the MLP is going to be used for. It is important to note that MLPs do not simply learn the associations between given input patterns and the corresponding outputs, but they really learn the general non–linear law, expressed as a combination of non–linear sigmoid functions, which connect a certain kind of inputs with a certain kind of outputs. This ability of MLPs to find a general relation between inputs and outputs starting from a relatively small amount of sample patterns is very useful for function approximation, pattern classification and modeling of unknown systems.

The major drawback of MLPs is that neither the number of neurons in the hidden layers nor the exact topology of these layers, needed to provide a certain approximation, is known in advance: only the number of neurons in the input and output layers are known, which are given by the dimensions of the input and output sets, i.e. *N* for the input layer and *M* for the output, respectively.

Nevertheless, it has been proved by Hornik in 1991 [S13] that MLPs with just three layers (i.e. the input layer, a hidden layer and the output layer) can approximate any function f: R^N^ −→ R^M^ up to any desired precision, and that the obtained precision depends only on the number of neurons in the hidden layer. In other words, MLPs are universal approximators on a compact subset of R^N^, and this approximation can be performed by a simple three-layer network with a sufficient number of neurons in the hidden layer.

We describe here how The Multi-Layer Perceptron classifier has been trained and tested to discriminate the DD strategy from other strategies. The classifier for each frequency band has three inputs, one for each of the three measures considered (efficiency, divisibility and modularity), and two outputs. The inputs are the values of the Z–scores, $Z^{k}(D)$, and $Z^{k}(Q)$ and the output of the classifier is (1, 0) if the pattern corresponds to a DD network and (0, 1) otherwise. About 80% of the available patterns have been used for training, and the remaining 20% have been used for validation. The accuracy of the classifiers obtained for each of the four frequency bands are of 90%, 73%, 88% and 80% for Theta, Alpha, Beta and Gamma respectively.

The on–line classification of DD trials by means of Multi–Layer Perceptron requires only the computation of modularity, divisibility and efficiency (constant time complexity), and the feed of Z–scores to the MLPs, which corresponds to two matrix multiplications.

# Supporting References

S11 Cybenko, G. Approximation by superpositions of a sigmoidal function. *Mathematics of Control, Signals, and Systems (MCSS)*, **2(4)**:303–314 (1989).

S12 Haykin, S. Neural Networks: A Comprehensive Foundation (2 ed.). Prentice Hall. ISBN 0132733501 (1998).

S13 Hornik, K. Approximation Capabilities of Multilayer Feedforward Networks. *Neural Networks* **4**, (1991).
