# Supplementary material for: Defecting or Not Defecting: How to “Read” Human Behavior during Cooperative Games by EEG Measurements
Source: PLoS One. 2010 Dec 1;5(12):e14187. doi: 10.1371/journal.pone.0014187 (PMC2995728; doi:10.1371/journal.pone.0014187)
Supplement: Methods S3 — (0.04 MB DOC) [file pone.0014187.s003.doc]

# Methods S3

### High-resolution EEG

High-resolution EEG technologies have been developed to enhance the poor spatial information content of the EEG activity [S1-S5]. In this work, cortical activity from EEG scalp recordings is estimated by using a realistic geometry head model. This model consists of about 5,000 dipoles uniformly disposed on the cortical surface gathered from MRI images. The estimation of the current density strength for each dipole is obtained by solving the linear inverse problem according to techniques described in previous works [S6] and illustrated in the following. The solution of the following linear system:

| Ax = b + n | (S1) |
| --- | --- |

provides an estimation of the dipole source configuration **x** that generates the measured EEG potential distribution **b**. The system also includes the measurement of a noise **n**, which is assumed to have a normal distribution [S1; S7]. **A** is the lead field matrix, where each *j*-th column describes the potential distribution generated on the scalp electrodes by the *i*-th unitary dipole. The current density solution vector **ξ** of Eq. (S1) was obtained as [S7; S4]:

| $\xi=\mathop{\mathrm{argmin}}\limits_{x} \left(\left\Vert\mathbf{A}x-b\right\Vert_{\mathbf{M}}^{2}+\lambda^{2}\left\Vert x\right\Vert_{\mathbf{N}}^{2}\right)$ | (S2) |
| --- | --- |

where **M**, **N** are the matrices associated respectively to the metrics of the data and of the source space, λ is the regularization parameter and || x ||_M_ represents the *M*-norm of vector **x**. The solution of Eq. (S2) is given by the inverse operator **G**:

| $\mathbf{\xi}\left(t\right)=\mathbf{Gb}\left(t\right)$, | (S3) |
| --- | --- |

An optimal regularization of this linear system was obtained by the *L*-curve approach [S3]. As metric in the data space we used the identity matrix, while as norm in the source space we use the following metric

| $\left(\mathbf{N}^{-1}\right)_{ii}=\left\Vert\mathbf{A}_{\cdot i}\right\Vert^{-2}$ | (S4) |
| --- | --- |

where (**N**^-1^)_ii_ is the i-th element of the inverse of the diagonal matrix **N** and all the other matrix elements N_ij_ are set to 0. The L_2_ norm of the *i*-th column of the lead field matrix **A** is denoted by||**A_.i_**||**.**

The method described above allows estimating over time the magnitude of the dipolar moment, as well as its sign, for each cortical dipole. Since in the head model we use, the orientation of the dipole is perpendicular to the cortical surface, this mathematical approach returns a scalar rather than a vector field. Then, at each time point, the spatial average of the magnitude derived from all the dipoles corresponding to a particular ROI is used to estimate **ρ**(*t*), i.e. the waveforms of the cortical activity in that ROI.

The spatial average process can be expressed in terms of the application of a matrix **T** to the cortical current density waveforms**.** This matrix is sparse and it has as many rows as ROIs, and as many columns as the number of dipole sources. ROI cortical current density waveforms can then be expressed as:

| **ρ**(*t*) = **Tx**(*t*) = **TGb**(*t*) = **G**_ROI_**b**(*t*) , **G**_ROI_ = **TG** | (S5) |
| --- | --- |

where **b**(t) is the array of the waveforms recorded from the scalp electrodes and **x**(t) is the array of the cortical current density waveforms estimated at the cortical surface. The **G_ROI_** matrix only depends on the volume conductor geometrical factor, and can thus be computed and stored off-line. The application of **G**_ROI_ to vector **b**(*t*) can be interpreted as a spatial filtering of the scalp potential using the elements of **G**_ROI_ as weights. Finally, time-varying waveforms at the level of different cortical areas are obtained.

# Supporting References

S1. Nunez, P. L. Neocortical dynamics and human EEG rhythms New York: Oxford University Press. 1995

S2. He, B., Wang, Y. and Wu, D. Estimating cortical potentials from scalp EEG's in a realistically shaped inhomogeneous head model by means of the boundary element. method, IEEE Trans. Biomed. Eng., vol. 46, pp. 1264-1268, Oct. (1999).

S3. Dale, A.M., Sereno, M.I. (1993) Improved localization of cortical activity by combining EEG and MEG with MRI cortical surface reconstruction: A linear approach. *Journal of Cognitive Neuroscience*, 5 (2), pp. 162-176.

S4 Babiloni, F. et al. Estimation of the cortical functional connectivity with the multimodal integration of high resolution EEG and fMRI data by Directed Transfer Function, *Neuroimage*, **24**(1):118-31 (2005).

S5. Cuspineda Bravo, E.R., Machado, C., Virues, T., Martínez-Montes, E., Ojeda, A., Valdés, P.A., Bosch, J., Valdes, L. Source analysis of alpha rhythm reactivity using LORETA imaging with 64-channel EEG and individual MRI (2009) Clinical EEG and Neuroscience, 40 (3), pp. 150-156.

S6 De Vico Fallani F, Astolfi L, Cincotti F, Mattia D, Tocci A, Marciani MG, Colosimo A, Salinari S, Gao S, Cichocki A and Babiloni F. Extracting Information from Cortical Connectivity Patterns Estimated from High Resolution EEG Recordings: A Theoretical Graph Approach. *Brain Topography*, 19(3):125-36, (2007).

S7 Grave de Peralta Menendez, R. and Gonzalez Andino, S.L. Distributed source models: standard solutions and new developments. In Uhl, C. (ed.). Analysis of neurophysiological brain functioning, Springer Verlag, pp. 176-201 (1999).
