# Supplementary material for: Defecting or Not Defecting: How to “Read” Human Behavior during Cooperative Games by EEG Measurements
Source: PLoS One. 2010 Dec 1;5(12):e14187. doi: 10.1371/journal.pone.0014187 (PMC2995728; doi:10.1371/journal.pone.0014187)
Supplement: Methods S4 — (0.03 MB DOC) [file pone.0014187.s004.doc]

# Methods S4

### Hyper-brain

One assumption in the MVAR methods when used to estimate the causality relationships between different waveforms is that the same “noise” characteristic for each MVAR model has to be used for the simultaneous estimation of the coefficient from the different waveforms recorded. However, it is not mathematically possible to simply extend the set of equations described above for a single case in the estimation of causality for two or more subjects’. The reason lies in the fact that the properties of the EEG of the different subjects could show a large deviation from the subject to subject. A normalization procedure is needed for the estimation of the causality between the multi-subjects MVAR. In this respect, the statistical treatment of each subject consisted in the z-score transformation of the original cortical signals by removing their mean and by standardizing their variance. In such a way, the possible inter-differences between the functional activities of two separate brains are minimized, while preserving at the same time the characteristic intra-differences among the functional activities in each single brain.

The implementation of the hyper-methods to compute the connectivity between different subjects has been performed by generating a unique Multivariate Autoregressive Model based on the EEG data from the two subjects belonging to the same team (i.e., in this case, the first and the second players). We applied PDC algorithms for the analysis of connectivity patterns within each brain, as well as for the hyper-connectivity links between subjects, on the z-transformed estimated cortical waveforms.
