# Supplementary material for: Defecting or Not Defecting: How to “Read” Human Behavior during Cooperative Games by EEG Measurements
Source: PLoS One. 2010 Dec 1;5(12):e14187. doi: 10.1371/journal.pone.0014187 (PMC2995728; doi:10.1371/journal.pone.0014187)
Supplement: Methods S5 — (0.05 MB DOC) [file pone.0014187.s005.doc]

# Methods S5

### MVAR models

The estimation of the functional connectivity between cortical areas during the tasks examined was assessed by using the Partial Directed Coherence (PDC) [S8]. PDC is a full multivariate spectral measure, used to determine the directed influences between any given pair of signals in a multivariate data set. It is computed on a Multivariate Autoregressive model (MVAR) that simultaneously models the whole set of signals. PDC is based on the concept of Granger causality [S9], according to which an observed time series *x(n)* is said to cause another series *y(n)* if the prediction error for *y(n)* at the present time is reduced by the knowledge of *x(n)*’s in the past, being *n* the single sample of data. This kind of relation is not reciprocal, thus allowing for the determination of the direction of information flow between signals.

Let Y(t) = [y_1_ (t), y_2_ (t), …, y_N_ (t)]^T^ be a set of cortical waveforms obtained from *N* different ROI and measured over time *t*. We suppose that the following MVAR process is an adequate description of the data set *Y(t)*:

| with Λ(0) = I | (S7) |
| --- | --- |

where **E**(t)=[e_1_(t), …, e_N_]^T^ is a vector of multivariate zero-mean uncorrelated white noise processes, Λ(1), Λ(2), … Λ(p) are the NxN matrices of model coefficients and *p* is the order of the MVAR mode. In the present study, *p* was chosen by means of the Akaike Information Criteria (AIC) for MVAR processes and was used for fitting to simulations, as well as to experimental signals. Although usually the sensitivity of MVAR performance depends on the model order, in our study small changes to the model order do not affect the results obtained [S4]. A modified procedure for fitting of MVAR on multiple trials has been adopted [S10]. Once a MVAR model is adequately estimated, it becomes the basis for subsequent spectral analysis. To investigate the spectral properties of the examined process, Eq. (S7) is transformed to the frequency domain:

| **Λ**(f) **Y**(f) = **E**(f) | (S8) |
| --- | --- |

where:

|  | (S9) |
| --- | --- |

and Δt is the temporal interval between two samples. Eq. (S8) can be rewritten as:

| Y(f) = Λ^-1^(f) E(f) = H(f) E(f). | (S10) |
| --- | --- |

*H(f)* is the transfer matrix of the system, whose element H_ij_ represents the connection between the j-th input and the i-th output of the system.

Partial coherence is an estimator of the relationship between a pair of signals, describing the interaction between the i-th ROI and j-th ROI when the influence due to all *(N-2)* time series is discounted it is defined by the formula:

|  | (S11) |
| --- | --- |

where *M_ij_(f)* is the minor obtained by removing the *i*-th row and the *j*-th column from the spectral matrix *Λ*(f). In [S8], Baccalà & Sameshima proposed for *χ_ij_(f)* the following factorisation:

|  | (S12) |
| --- | --- |

where is the *n*-th column of the matrix . This led to the definition of Partial Directed Coherence [S7]:

|  | (S13) |
| --- | --- |

The PDC from the j-th ROI to i-th ROI, π_ij_(f), describes the directional flow of information from the activity in the j-th ROI, s_j_(n), to the activity in the i-th ROI, s_i_(n), whereupon common effects produced by the other ROIs s_k_(n) on the latter are subtracted, leaving only a description that is specifically from s_j_(n) to s_i_(n).

PDC values are in the interval [0, 1], and the normalization condition

|  | (S14) |
| --- | --- |

is verified. According to this condition, *π_ij_(f)* represents the fraction of the time evolution of ROI *j* directed to ROI *i*, compared to all of *j*’s interactions with other ROIs.

# Supporting References

S4 Babiloni, F. et al. Estimation of the cortical functional connectivity with the multimodal integration of high resolution EEG and fMRI data by Directed Transfer Function, *Neuroimage*, **24**(1):118-31 (2005).

S8 Baccalà, L.A. and Sameshima, K. Partial Directed Coherence: a new concept in neural structure determination. *Biol Cybern*, **84**: 463-474, (2001).

S9. Granger, C.W.J. Investigating causal relations by econometric models and cross-spectral methods. *Econometrica*, 37p. 424-8 (1969).

S10. Ding, M., Bressler, S.L., Yang, W. and Liang Ding, H. Short-window spectral analysis of cortical event-related potentials by adaptive multivariate autoregressive modeling: Data preprocessing, model validation, and variability assessment. *Biol. Cybern*. **83**:35-45 (2000).
