# Supplementary figures and images for: Defecting or Not Defecting: How to “Read” Human Behavior during Cooperative Games by EEG Measurements
Source: PLoS One. 2010 Dec 1;5(12):e14187. doi: 10.1371/journal.pone.0014187 (PMC2995728; doi:10.1371/journal.pone.0014187)

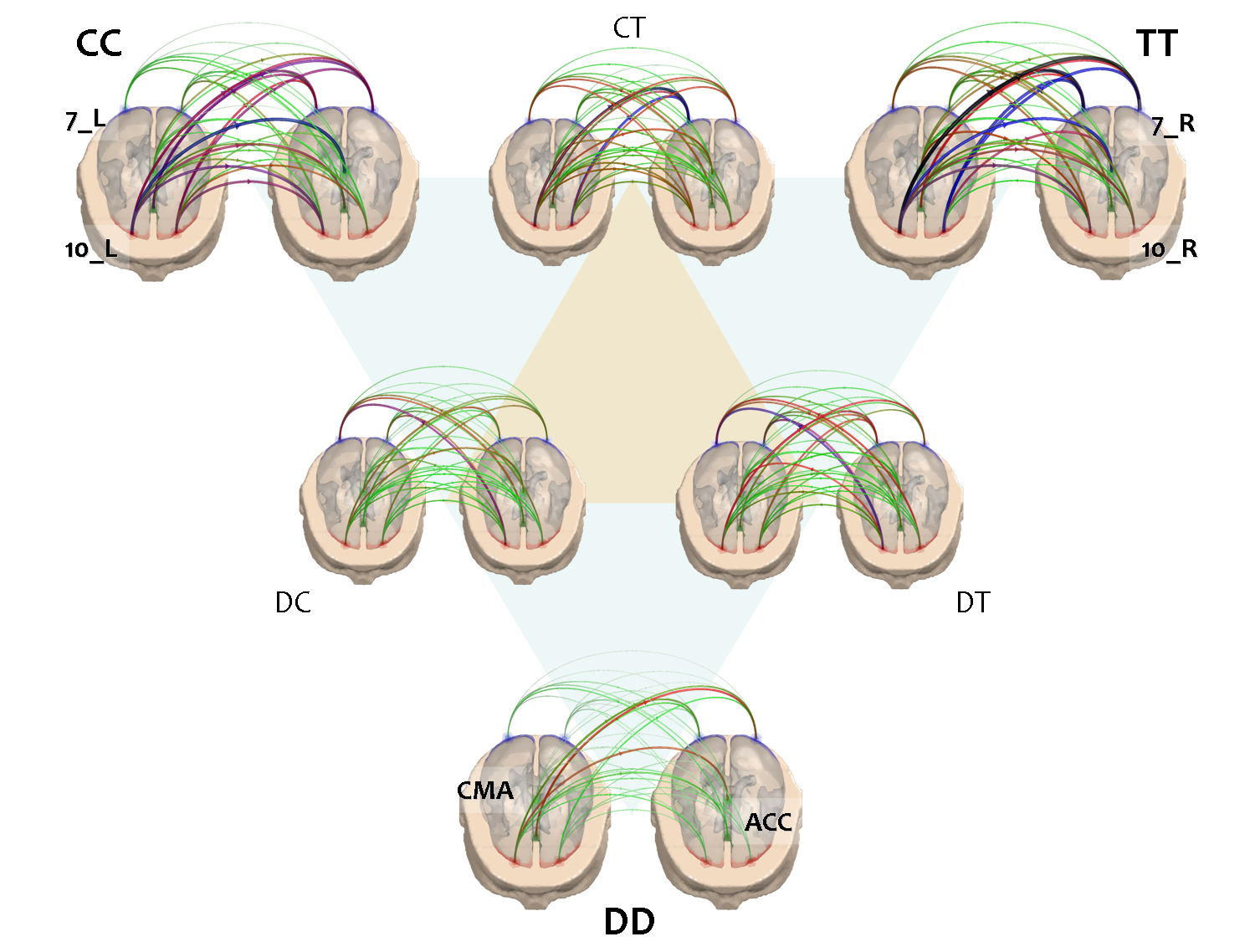

Supplement: Figure S1 — Inter-brain communication during pure and mixed strategies in the Alpha band. Two generic players are represented by the realistic head models used to estimate the cortical activity in the same six regions of interest (ROIs). Different colored points indicate the barycenters of these ROIs on the semi-transparent cortex. For the sake of simplicity, we didn't label the ROIs of each subplot, but just two for the CC (7_L, 10_L), TT (7_R, 10_R) and DD (CMA, ACC) subplot. Only links between the two brains are illustrated in each hyper-brain network, i.e. the inter-brain communication. The size and the color of each directed connection represent the average of the PDC values from all the 26 couples of subjects in the Alpha (8–13 Hz) frequency band. (1.06 MB TIF) [file pone.0014187.s006.tif]

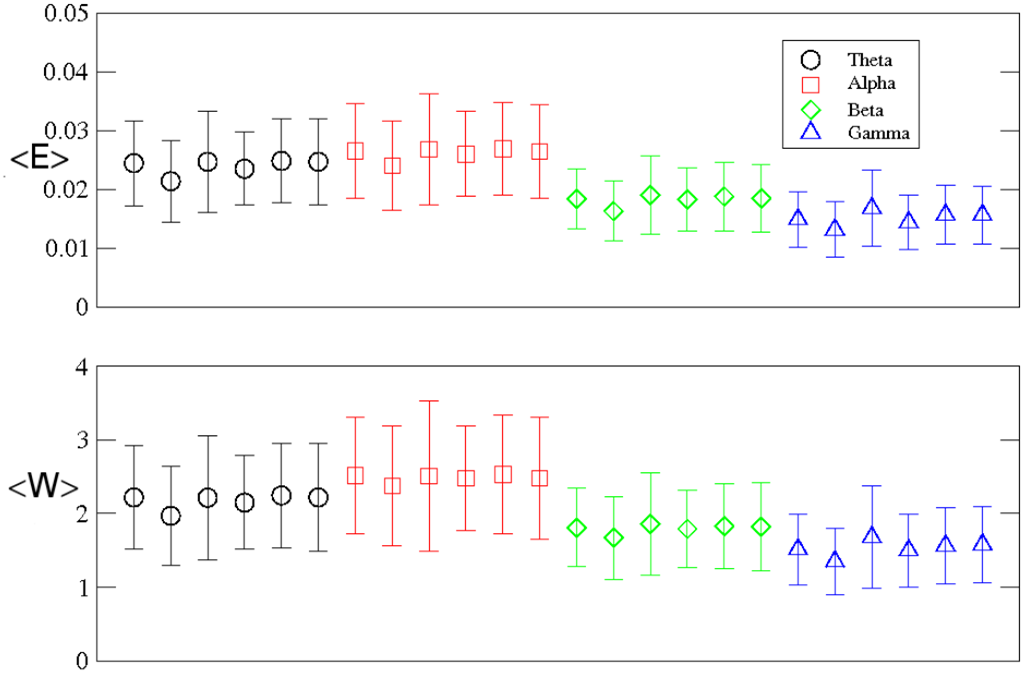

Supplement: Figure S2 — Average values and standard deviations of efficiency E (top) and total weight W (bottom), for CC, DD, TT, CD, CT, DT strategies (from left to right), respectively. Different bands are represented with different symbols and colors: Theta 4–7 Hz (black circles), Alpha 8–13 Hz (red squares), Beta 14–29 Hz (green diamonds) and Gamma 30–40 Hz (blue triangles). Averages and standard deviations are performed over the 26 couples of subjects. (0.12 MB TIF) [file pone.0014187.s007.tif]

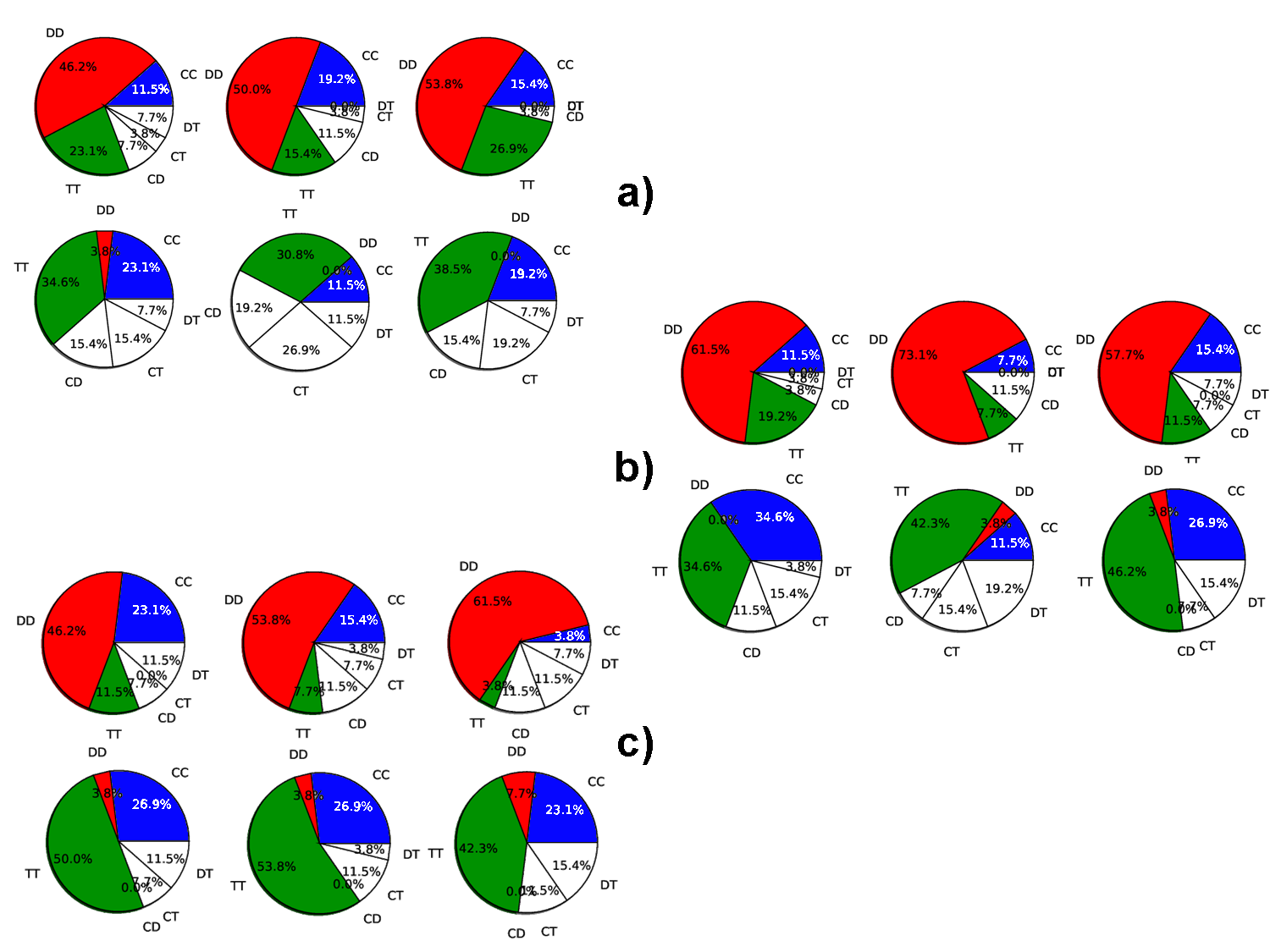

Supplement: Figure S3 — Pie diagrams of efficiency E, divisibility D and modularity Q relative to the (a) Alpha, (b) Beta and (c) Gamma band. Top panels: from left to right the diagrams represent the percentage of cases - over the 26 couples - in which graph efficiency E is minimal, whilst the divisibility D and modularity Q are maximal. Bottom panels: percentage of cases - over the 26 couples - in which E is maximal and D and Q are minimal. Blue areas represent pure cooperation CC, red areas represent pure defection DD, green areas represent pure tit-for-tat TT. Mixed situations CD, CT, and DT are represented by white areas. (0.37 MB TIF) [file pone.0014187.s008.tif]

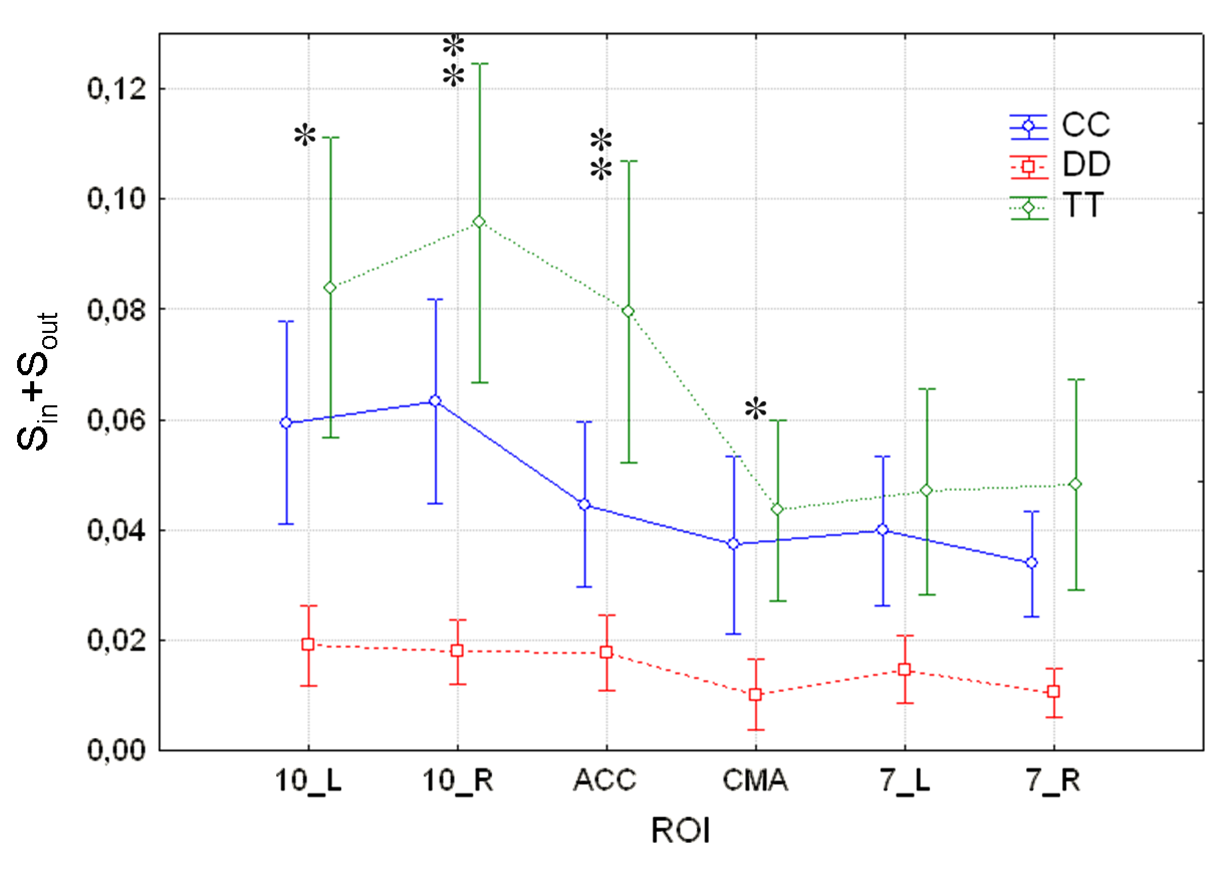

Supplement: Figure S4 — Values of total strength sin+sout for the six ROIs, labelled as in the horizontal axis (Gamma frequency band). Each line corresponds to a different task: CC (blue circles), DD (red squares) and TT (green diamonds). Vertical bars denote 0.95 confidence intervals. Single stars indicate the ROI where the DD strategy is significantly different (p<0.001) from the CC and from the TT strategy. Double stars mark the ROIs where all the three strategies are significantly different (p<0.001). (0.24 MB TIF) [file pone.0014187.s009.tif]

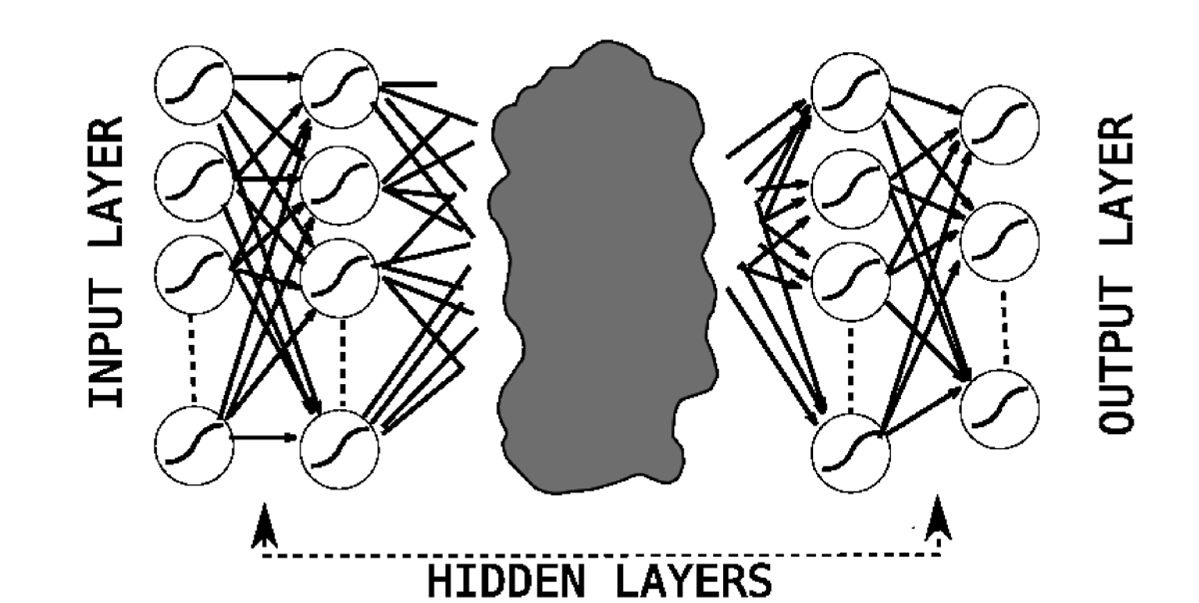

Supplement: Figure S5 — A schematic representation of a Multilayer Perceptron (0.21 MB TIF) [file pone.0014187.s010.tif]

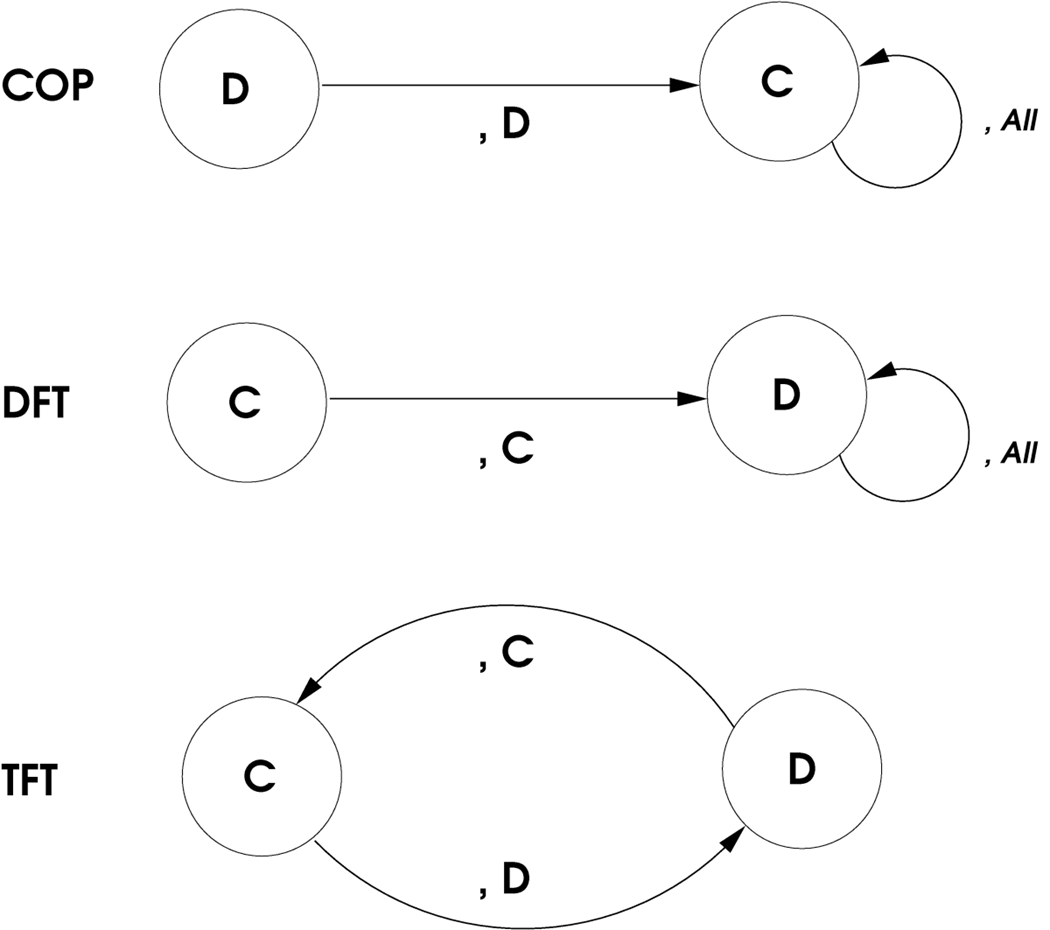

Supplement: Figure S6 — The three strategies of the Iterated Prisoner's Dilemma: Cooperation (top), Defection (middle) and Tit-for-Tat (bottom). (0.08 MB TIF) [file pone.0014187.s011.tif]
