# Supplementary material for: Defecting or Not Defecting: How to “Read” Human Behavior during Cooperative Games by EEG Measurements
Source: PLoS One. 2010 Dec 1;5(12):e14187. doi: 10.1371/journal.pone.0014187 (PMC2995728; doi:10.1371/journal.pone.0014187)
Supplement: Table S1 — (0.08 MB DOC) [file pone.0014187.s012.doc]

# Table S1

Averages and standard deviations for the standard network measures computed over the 26 samples in each frequency band.

| Trials |  | Edges *K* | MaxOutDeg | TotalWeight *W* | MaxOutStrength | Efficiency *E* | Clustering *C* |
| --- | --- | --- | --- | --- | --- | --- | --- |
| **CC** | Theta | 51.8±9.1 | 6.8±1.1 | 2.2±0.7 | 0.36±0.14 | 0.024±0.007 | 0.58±0.12 |
|  | Alpha | 53.2 ±12.0 | 6.8 ±1.5 | 2.5 ±0.8 | 0.42 ±0.14 | 0.027 ±0.008 | 0.61 ±0.12 |
|  | Beta | 51.3 ±12.5 | 6.8 ±1.7 | 1.8 ±0.5 | 0.31 ±0.10 | 0.019 ±0.005 | 0.60 ±0.16 |
|  | Gamma | 46.0 ±11.0 | 7.3 ±1.7 | 1.5 ±0.5 | 0.31 ±0.10 | 0.015 ±0.005 | 0.44 ±0.17 |
| **DD** | Theta | 44.4±9.2 | 6.0 ±1.5 | 1.97±0.7 | 0.36±0.16 | 0.021±0.007 | 0.59±0.12 |
|  | Alpha | 47.8 ±  8.5 | 6.0 ±1.1 | 2.4 ±0.8 | 0.42 ±0.15 | 0.024 ±0.008 | 0.66 ±0.12 |
|  | Beta | 44.7± 9.2 | 5.5 ±1.1 | 1.7 ±0.6 | 0.32 ±0.13 | 0.016 ±0.005 | 0.63 ±0.14 |
|  | Gamma | 37.6 ±8.5 | 5.7 ±1.6 | 1.4 ±0.5 | 0.30± 0.1 | 0.013 ±0.005 | 0.48 ±0.16 |
| **TT** | Theta | 52.0±12.6 | 7.0±1.7 | 2.2±0.8 | 0.40±0.16 | 0.025±0.008 | 0.55±0.13 |
|  | Alpha | 55.4 ±13.6 | 7.2 ±1.8 | 2.5 ±1.0 | 0.41 ±0.15 | 0.027 ±0.009 | 0.61 ±0.14 |
|  | Beta | 53.6 ±15.2 | 6.7 ±1.7 | 1.9 ±0.7 | 0.31 ±0.11 | 0.019 ±0.007 | 0.61 ±0.12 |
|  | Gamma | 48.1 ±14.3 | 7.3 ±1.8 | 1.7 ±0.7 | 0.34 ±0.13 | 0.017 ±0.005 | 0.48 ±0.17 |
| **CD** | Theta | 49.7±8.2 | 7.6 ±1.6 | 2.14 ±0.7 | 0.48± 0.20 | 0.023 ±0.007 | 0.47± 0.13 |
|  | Alpha | 53.0 ±  9.1 | 7.9 ±1.6 | 2.5 ±0.7 | 0.57 ±0.21 | 0.026 ±0.007 | 0.55 ±0.11 |
|  | Beta | 48.7 ±12.0 | 8.0 ±1.9 | 1.8 ±0.6 | 0.43 ±0.16 | 0.018 ±0.006 | 0.53 ±0.14 |
|  | Gamma | 42.5 ±11.8 | 7.0 ±2.1 | 1.5 ±0.5 | 0.38 ±0.22 | 0.014 ±0.005 | 0.41 ±0.13 |
| **CT** | Theta | 52.8 ±10.7 | 8.1 ±1.6 | 2.24± 0.7 | 0.51 ±0.21 | 0.024 ±0.007 | 0.48 ±0.12 |
|  | Alpha | 56.0 ±11.1 | 8.5 ±1.3 | 2.5 ±0.8 | 0.60 ±0.21 | 0.027 ±0.008 | 0.51 ±0.11 |
|  | Beta | 52.0 ±12.7 | 8.3 ±1.8 | 1.8 ±0.6 | 0.44 ±0.17 | 0.019 ±0.006 | 0.52 ±0.12 |
|  | Gamma | 46.8 ±13.0 | 7.9 ±1.9 | 1.6 ±0.5 | 0.41 ±0.18 | 0.016 ±0.005 | 0.40 ±0.13 |
| **DT** | Theta | 52.6 ±10.8 | 8.0 ±1.6 | 2.2 ±0.8 | 0.51 ±0.23 | 0.025 ±0.007 | 0.47±0.12 |
|  | Alpha | 55.1 ±11.6 | 8.1 ±1.7 | 2.5 ±0.8 | 0.54 ±0.24 | 0.027 ±0.008 | 0.52 ±0.13 |
|  | Beta | 52.0 ±12.0 | 8.1 ±2.0 | 1.8 ±0.6 | 0.43 ±0.18 | 0.019 ±0.006 | 0.53 ±0.15 |
|  | Gamma | 46.5 ±12.7 | 7.6 ±1.7 | 1.6 ±0.5 | 0.41 ±0.21 | 0.016 ±0.005 | 0.40 ±0.14 |
