# Supplementary material for: Defecting or Not Defecting: How to “Read” Human Behavior during Cooperative Games by EEG Measurements
Source: PLoS One. 2010 Dec 1;5(12):e14187. doi: 10.1371/journal.pone.0014187 (PMC2995728; doi:10.1371/journal.pone.0014187)
Supplement: Table S2 — (0.03 MB DOC) [file pone.0014187.s013.doc]

# Table S2

ANOVA effects of the independent task factors TASK and ROI. The * indicates a significance of *p*<0.001 for the interaction factor TASK x ROI.

| Bands | Task | ROI | Task x ROI |
| --- | --- | --- | --- |
| Theta | *F*(2,102)=15*.*3 *p*<0.000001 | *F*(5,255)=4.7 *p*=0.00039 | *F*(10,510)=0.9 *p*=0.51226 |
| Alpha | *F*(2,102)=17*.*8 *p*<0.000001 | *F*(5,255)=3.7 *p*=0.00272 | *F*(10,510)=0.8 *p*=0.64233 |
| Beta | *F*(2,102)=20.1 *p*<0.000001 | *F*(5,255)=9.0 *p*<0.000001 | *F*(10,510)=3.2 *p*=0.00057 ***** |
| Gamma | *F*(2,102)=25.2 *p*<0.000001 | *F*(5,255)=7.5 *p*<0.000001 | *F*(10,510)=3.8 *p*=0.00005 ***** |
